# Therapeutic Potential of Bioactive Peptides Derived from Natural Products of Tortoiseshell and Antler in Alleviating Osteoporosis and Osteoarthritis

**DOI:** 10.3390/ijms26020581

**Published:** 2025-01-11

**Authors:** Kou-Toung Chung, Hsuan-Mei Wu, Ming-Chung Lee, Wu-Chang Chuang, Chung-Hsin Wu

**Affiliations:** 1 Department of Health Nutrition and Chemical Engineering, Army Academy of ROC, Taoyuan City 320, Taiwan; 2 School of Life Science, National Taiwan Normal University, Taipei 117, Taiwan; mandywu0906@gmail.com; 3 Brion Research Institute of Taiwan, New Taipei City 231, Taiwan; mileslee@sunten.com.tw (M.-C.L.); cwctd331@sunten.com.tw (W.-C.C.)

**Keywords:** traditional Chinese medicines, tortoiseshell, antler, bioactive peptides, osteoporosis, osteoarthritis, natural products

## Abstract

Tortoiseshell and antler, the main components of *Guilu Erxian Jiao*, are natural products that can be used as traditional Chinese medicine (TCM) to alleviate osteoporosis and osteoarthritis. However, research on the active ingredients in tortoiseshell and antler for alleviating osteoporosis and osteoarthritis remains insufficient. This study primarily compares the antioxidant capacity of tortoiseshell gelatin and antler gelatin and their bioactive peptides, as well as their effects on the cell viability of MC3T3-E1 osteoblasts and HIG-82 chondrocytes. Our results indicate that when tortoiseshell and antler are combined with their respective bioactive peptides, the antioxidant capacity and cell viability of osteoblasts and chondrocytes are superior to the effects of either used alone. Comparing the antioxidant capacity and cell viability of osteoblasts and chondrocytes of tortoiseshell and antler and their respective bioactive peptides when used alone, we found that tortoiseshell and its bioactive peptides have better cell viability and calcium deposition for osteoblasts compared to the antler and its bioactive peptides. Conversely, antler and its bioactive peptides exhibit stronger antioxidant capacity and better cell viability for chondrocytes than tortoiseshell and its bioactive peptides. These results suggest that the alleviation of osteoporosis may mainly be attributed to tortoiseshell and its bioactive peptides, while the alleviation of osteoarthritis may mainly be attributed to antler and its bioactive peptides. When tortoiseshell and antler are used together, they can provide the best therapeutic effects for both osteoporosis and osteoarthritis.

## 1. Introduction

The traditional Chinese medicine (TCM) of *Guilu Erxian Jiao* (GEJ) is a composed of tortoiseshell, antler, wolfberry, and ginseng, which are primarily used to alleviate degenerative joint diseases, osteoporosis, osteoarthritis, and joint pain [1]. Previous studies have indicated that GEJ can enhance osteoblast differentiation markers and increase the production of bone morphogenetic proteins through the PI3K/Akt/NF-κB signaling pathway to treat osteoporosis [2]. Additionally, clinical studies have found that GEJ can effectively alleviate joint pain and improve joint inflammation [1,3]; effectively relieve muscle degeneration and muscle strength decline [4]; and treat postmenopausal osteoporosis in women. Our previous research also found that GEJ can alleviate the adverse effects of cancer chemotherapy, alleviating weight loss, motor dysfunction, blood circulation abnormalities, bone marrow suppression, myocardial damage, joint degeneration, and osteoporosis caused by cancer chemotherapy in mice [5].

Tortoiseshell, with its neutral properties, has effects such as nourishing yin, reducing fire, tonifying the kidneys, strengthening bones, nourishing blood, and calming the heart. It has the function of nourishing yin and subduing yang [6]. Pharmacological studies have shown that turtle shell extract can improve the immune system [7]. Tortoiseshell is not only an anti-aging food [8,9] but also protects nerves from degeneration [10]. Modern pharmacological research indicates that tortoiseshell has the ability to enhance immune function, has anti-tumor effects, benefits the kidneys, and strengthens bones. Currently, it is commonly used to treat symptoms such as night sweats and seminal emission caused by kidney yin deficiency, as well as muscle and bone weakness in children [11,12]. Furthermore, antler has been documented in classical TCM for over 2000 years and is believed to have effects such as nourishing yin, tonifying the kidneys, strengthening the spleen, fortifying muscles and bones, and promoting blood circulation [13]. Pharmacological studies have found that antler glue has activities such as immunomodulation, anti-cancer, anti-fatigue, anti-osteoporosis, anti-inflammatory, analgesic, antibacterial, antiviral, anti-stress, antioxidant, and hypoglycemic effects, and hematopoietic regulation, and it also has therapeutic effects on mammary gland hyperplasia [13]. Although its mechanism of action is not yet clear, its pharmacological activity is likely mainly attributed to its primary bioactive components, including amino acids, peptides, and proteins. According to animal studies and clinical trials, antler glue does not cause severe side effects.

We feel that the current research on the active ingredients in tortoiseshell and antler for alleviating osteoporosis and osteoarthritis is insufficient. This study primarily examines the role of tortoiseshell, antler, and their combination (*Guilu Jiao*, GJ), in alleviating osteoporosis and osteoarthritis. The GJ is an extract made from a combination of tortoiseshell and antler. We compared the antioxidant capacities of GJ, tortoiseshell and antler, as well as their effects on the viability of MC3T3-E1 osteoblasts and HIG-82 chondrocytes. Additionally, we isolated various bioactive peptides from GJ, tortoiseshell and antler gelatin and then compared their antioxidant capacities and effects on the viability of HIG-82 chondrocytes. We hope that our results can provide a basis for developing GJ as a natural product for alleviating osteoporosis and osteoarthritis.

## 2. Results

### 2.1. Antioxidant Capacity of Tortoiseshell, Antler, and the Combination of Tortoiseshell and Antler

**Figure 1:**
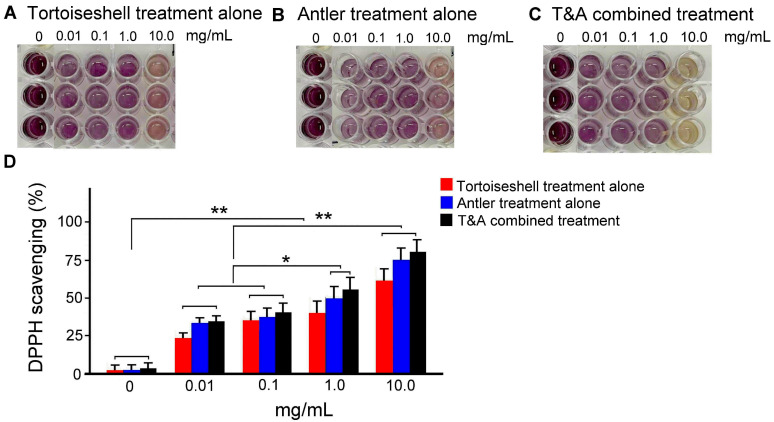
Comparison of antioxidant capacity of tortoiseshell, antler, and the combination of tortoiseshell and antler. (**A**–**C**) DPPH radical scavenging assay to compare the antioxidant capacity of tortoiseshell (**A**), antler (**B**), and their combination (**C**) in scavenging DPPH radicals. (**D**) Quantitative analysis of scavenging DPPH radicals among tortoiseshell, antler, and their combination. T&A combined treatment: tortoiseshell and antler combined treatment. Data are shown as mean ± SEM, N = 3 for each treatment. ** *p* < 0.01 and * *p* < 0.05, two-way ANOVA followed by Student–Newman–Keuls multiple-comparison posttest.

First, we used the DPPH radical scavenging assay to compare the antioxidant capacity of tortoiseshell, antler, and the combination of tortoiseshell and antler in scavenging DPPH radicals (Figure 1A–C). The results showed that, compared to 0.01 and 0.1 mg/mL, the radical scavenging rates of tortoiseshell, antler, and the combination of tortoiseshell and antler significantly increased with increasing concentration (Figure 1D, *p* < 0.01–0.05). These results indicate that tortoiseshell, antler, and the combination of tortoiseshell and antler all have good antioxidant and radical scavenging abilities. As shown in Figure 1D, at the same concentration, the combination of tortoiseshell and antler seems to have the best antioxidant and radical scavenging abilities, followed by antler, and tortoiseshell being slightly less effective than the other two. However, there was no statistically significant difference in the antioxidant and radical scavenging abilities at the same concentration among tortoiseshell, antler, and the combination of tortoiseshell and antler (Figure 1D, *p* > 0.05).

### 2.2. Effects of Tortoiseshell, Antler, and Their Combination on Mineralization in MC3T3-E1 Osteoblasts

**Figure 2:**
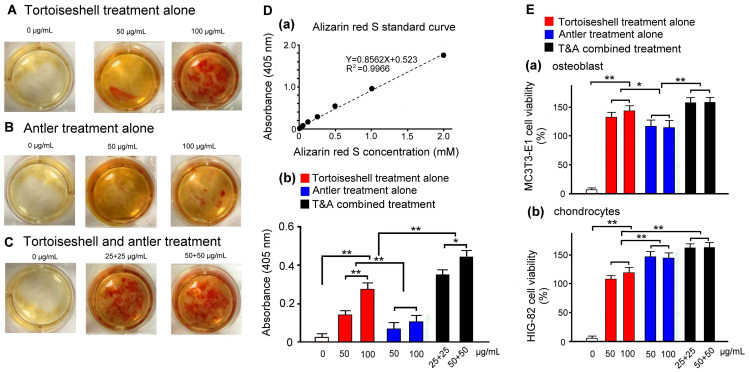
Effects of tortoiseshell, antler, and their combination on mineralization in MC3T3-E1 osteoblasts, and cell viability in MC3T3-E1 osteoblasts and HIG-82 chondrocytes. (**A**–**C**) Alizarin red S staining of MC3T3-E1 osteoblasts with individual treatments of tortoiseshell (**A**), antler (**B**), and tortoiseshell and antler (T&A) combined treatments (**C**). (**D**) Standard curve of alizarin red S concentration (**a**), and quantitative analysis of mineralization through alizarin red S staining in MC3T3-E1 osteoblasts with individual treatments of tortoiseshell, antler, and their combination (**b**). (**E**) Cell viability through MTT assay in MC3T3-E1 osteoblasts (**a**) and HIG-82 chondrocytes (**b**). Data are shown as mean ± SEM, N = 3 for each treatment. ** *p* < 0.01 and * *p* < 0.05, two-way ANOVA followed by Student–Newman–Keuls multiple-comparison posttest.

We evaluated the bioactivity of tortoiseshell, antler, and their combination on the calcium mineralization of MC3T3-E1 osteoblasts. By adding tortoiseshell, antler, and their combination individually to the culture medium and examining the calcium mineralization of MC3T3-E1 osteoblasts, we confirmed their entry into the mineral deposition stage. MC3T3-E1 osteoblasts cultured for 14 days were stained with alizarin red S to quantitatively and qualitatively measure cell calcium mineralization. Figure 2A–C show the effects of tortoiseshell, antler, and their combination on the mineralization of MC3T3-E1 cells. Alizarin red S staining showed slight red spots at the mineralization sites of MC3T3-E1 osteoblasts, with the combination of tortoiseshell and antler treatment showing the most intense color, indicating that the combination of tortoiseshell and antler effectively promotes the calcium mineralization of MC3T3-E1 cells. Next was the tortoiseshell-treated MC3T3-E1 osteoblasts, which showed a moderate color intensity, while the antler-treated MC3T3-E1 osteoblasts showed the weakest color intensity. These results suggest that when tortoiseshell and antler are treated individually, tortoiseshell treatment has a better effect on the calcium mineralization of MC3T3-E1 osteoblasts, while antler treatment is less effective.

Using the alizarin red S concentration standard curve in Figure 2D(a), Figure 2D(b) quantified the degree of calcium mineralization of MC3T3-E1 osteoblasts under different concentrations of tortoiseshell, antler, and their combination. The results showed that high concentrations of tortoiseshell, antler, and their combination significantly increased the degree of the calcium mineralization of MC3T3-E1 osteoblasts compared to low concentrations (*p* < 0.01–0.05). At the same concentration, the combination of tortoiseshell and antler showed the best performance, followed by tortoiseshell, and antler showed the weakest performance, with significant differences between them (*p* < 0.01). We speculate that in the combination of tortoiseshell and antler, tortoiseshell mainly promotes the binding of cells and calcium and should effectively promote the osteogenic differentiation of MC3T3-E1 osteoblasts.

### 2.3. Evaluation of Effects of Tortoiseshell, Antler, and Their Combination on the Cell Viability of MC3T3-E1 Osteoblasts and HIG-82 Chondrocytes

We assessed the cell viability of MC3T3-E1 osteoblasts and HIG-82 chondrocytes treated with tortoiseshell, antler, and their combination. MC3T3-E1 osteoblasts and HIG-82 chondrocytes were cultured for 14 days, followed by a 48-h treatment with tortoiseshell, antler, and their combination. The cell viability was then evaluated. Figure 2E(a) quantifies the cell viability of MC3T3-E1 osteoblasts treated with varying concentrations of tortoiseshell, antler, and their combination. The results indicate a significant increase in cell viability for all treatments, with the combination treatment showing the highest efficacy, followed by tortoiseshell, and then antler. Significant differences were observed between each treatment group (*p* < 0.01–0.05), suggesting that tortoiseshell primarily contributes to the enhanced growth of MC3T3-E1 osteoblasts in the combination treatment. Figure 2E(b) quantifies the cell viability of HIG-82 chondrocytes treated with varying concentrations of tortoiseshell, antler, and their combination. The results show a significant increase in cell viability for all treatments, with the combination treatment again showing the highest efficacy, followed by antler, and then tortoiseshell. Significant differences were observed between each treatment group (*p* < 0.01), indicating that antler primarily contributes to the enhanced growth of HIG-82 chondrocytes in the combination treatment. Our findings suggest that the combination of tortoiseshell and antler may play a crucial role in alleviating osteoporosis and osteoarthritis, with tortoiseshell being more effective for osteoporosis and antler for osteoarthritis.

### 2.4. LC/MS Fingerprint of Amino Acids from Tortoiseshell, Antler, and Their Combination

**Figure 3:**
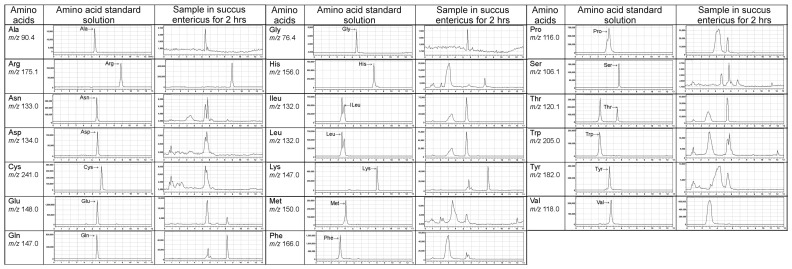
LC/MS fingerprint of amino acids from tortoiseshell, antler, and their combination after stirring in succus entericus for 2 h. Amino acids present in tortoiseshell and antler, as well as their combination, include alanine (Ala), arginine (Arg), asparagine (Asn), aspartic acid (Asp), cystine (Cys), glutamic acid (Glu), glutamine (Gln), glycine (Gly), histidine (His), isoleucine (Ile), leucine (Leu), lysine (Lys), methionine (Met), phenylalanine (Phe), proline (Pro), serine (Ser), threonine (Thr), tryptophan (Trp), tyrosine (Tyr), and valine (Val).

We conducted the identification and quantification of the marker components according to the pharmacopeia. We used high-performance liquid chromatography–mass spectrometry (LC/MS) to analyze the amino acid fingerprints of tortoise shell, deer antler, and their combination (Figure 3). Compared with amino acid standards, the results showed that the amino acids present in tortoise shell, deer antler, and their combination include alanine (Ala), arginine (Arg), asparagine (Asn), aspartic acid (Asp), cystine (Cys), glutamic acid (Glu), glutamine (Gln), glycine (Gly), histidine (His), isoleucine (Ile), leucine (Leu), lysine (Lys), methionine (Met), phenylalanine (Phe), proline (Pro), serine (Ser), threonine (Thr), tryptophan (Trp), tyrosine (Tyr), and valine (Val).

### 2.5. Antioxidant Capacity of Bioactive Peptides from Tortoiseshell, Antler, and the Combination of Tortoiseshell and Antler

**Figure 4:**
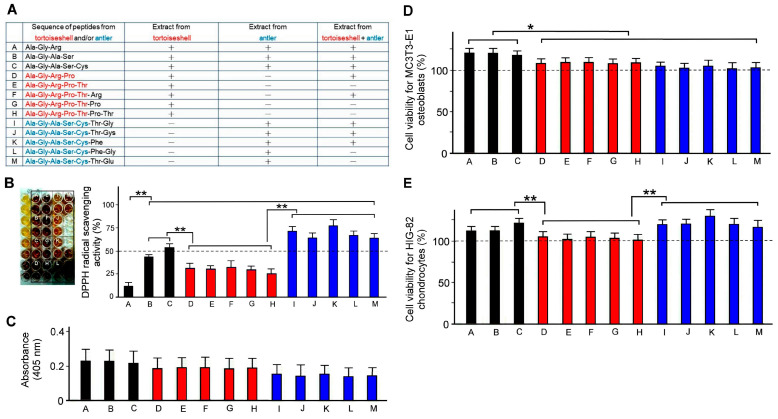
Bioactive peptides from tortoiseshell, antler, and their combination on DPPH free radical scavenging ability and the cell viability of HIG-82 chondrocytes. (**A**) Bioactive peptides (A~M) from tortoiseshell, antler, and their combination. The bioactive peptide sequences marked in red mainly come from tortoiseshell; the bioactive peptide sequences marked in blue mainly come from antler; the bioactive peptide sequences marked in black come from their combination. (**B**) Quantitative analysis of scavenging DPPH radicals with individual treatments among bioactive peptides (0.02 mg/mL for each) from tortoiseshell, antler, and their combination. (**C**) Quantitative analysis of mineralization through alizarin red S staining in MC3T3-E1 osteoblasts with individual treatments among bioactive peptides (0.02 mg/mL for each) from tortoiseshell, antler, and their combination. (**D**) Quantitative analysis of cell viability for MC3T3-E1 osteoblasts with individual treatments among bioactive peptides (0.02 mg/mL for each) from tortoiseshell, antler, and their combination through MTT assay. (**E**) Quantitative analysis of cell viability for HIG-82 chondrocytes with individual treatments among bioactive peptides (0.02 mg/mL for each) from tortoiseshell, antler, and their combination through MTT assay. Data are shown as mean ± SEM, N = 3 for each treatment. ** *p* < 0.01, * *p* < 0.05, one-way ANOVA followed by Student–Newman–Keuls multiple-comparison posttest.

We utilized succus entericus to hydrolyze tortoiseshell, antler, and their combination into short-chain peptides and analyzed the amino acid sequences of the bioactive peptides using LC-MS/MS. Our results showed that antler peptides are rich in Ala, Gly, Ser, Cys, Thr, and Glu, while tortoiseshell peptides are rich in Ala, Gly, Arg, Pro, and Thr. We sequenced the bioactive peptides hydrolyzed from tortoiseshell, antler, and their combination, identifying a total of 13 bioactive peptides, which were labeled A to M based on their different amino acid sequences (Figure 4A).

According to the amino acid sequences of the 13 bioactive peptides listed in Figure 4A, these peptides were synthesized and purified by a biotechnology company commissioned by the Brion Research Institute of Taiwan. The antioxidant capacity of these 13 bioactive peptides was tested using the DPPH radical scavenging assay. The experimental results showed that peptides B~C and I~M exhibited better DPPH radical scavenging activity (Figure 4B, *p* < 0.01). Since these bioactive peptides mostly contain the sequence fragment Ala-Ser-Cys and can all be found in the bioactive peptides hydrolyzed from antler, where the percentages of Ala, Ser, and Cys are the highest, we believe that Ala, Ser, and Cys should be a high-quality source of antioxidant bioactive peptides.

### 2.6. Effects of Bioactive Peptides from Tortoiseshell, Antler, and Their Combination on the Cell Viability of MC3T3-E1 Osteoblasts and HIG-82 Chondrocytes

We utilized the MTT assay to assess the effects of bioactive peptides from tortoiseshell, antler, and their combination on the viability of MC3T3-E1 osteoblasts and HIG-82 chondrocytes. Our results showed that the bioactive peptides A~C significantly enhanced the viability of MC3T3-E1 osteoblasts more than those of the bioactive peptides D~M (Figure 4D, *p* < 0.05); but the bioactive peptides A~C and I~M significantly enhanced the viability of HIG-82 chondrocytes more than those of the bioactive peptides D~H (Figure 4E, *p* < 0.01). As shown in Figure 4A, the bioactive peptides A~C were mainly hydrolyzed from the combination of tortoiseshell and antler, the bioactive peptides D~H were hydrolyzed from tortoiseshell, and the bioactive peptides I~M were hydrolyzed from antler. In Figure 4D, we found that the active peptides derived from the combination of tortoiseshell and antler significantly increased the cell viability of MC3T3-E1 osteoblasts compared to the active peptides derived individually from tortoiseshell or antler (*p* < 0.05). There was no significant difference in the cell viability of MC3T3-E1 osteoblasts among the active peptides derived individually from tortoiseshell and antler (*p* > 0.05). We evaluated the bioactive peptides from tortoiseshell, antler, and their combination on the calcium mineralization in MC3T3-E1 osteoblasts in Figure 4C. We found that the bioactive peptides from their combination appeared to induce a better calcium mineralization of MC3T3-E1 osteoblasts, followed by the bioactive peptides from tortoiseshell, and the bioactive peptides from antler showed the worse calcium mineralization of MC3T3-E1 osteoblasts. However, there was no statistical difference in the calcium mineralization of MC3T3-E1 osteoblasts among the active peptides derived individually from tortoiseshell and antler (*p* > 0.05). Comparing with the results in Figure 4B, we found that the effects of bioactive peptides from tortoiseshell, antler, and their combination on the cell viability of HIG-82 chondrocytes were very similar to their antioxidant capacity (Figure 4E). This indicates that the bioactive peptides from antler not only have strong antioxidant capacity but also significantly improve the viability of HIG-82 chondrocytes. In other words, the efficacy of GEJ in alleviating osteoarthritis may be attributed to the regenerative and reparative effects of antler bioactive peptides on chondrocytes.

## 3. Discussion

Bones provide structure and support to the body and store minerals such as calcium and phosphorus. Cartilage, primarily composed of chondrocytes, collagen, and proteoglycans, functions to support, protect, and assist in joint movement. Ancient Chinese medicine records indicate that GEJ can effectively treat osteoporosis and degenerative knee arthritis. However, scientific evidence from evidence-based medicine is still lacking. Our study found that when tortoiseshell and antler were used separately, they both significantly promoted the proliferation of MC3T3-E1 osteoblasts and HIG-82 chondrocytes, although the effects varied. For example, tortoiseshell was more effective in promoting the proliferation and calcium mineralization of MC3T3-E1 osteoblasts compared to antler. Conversely, antler was more effective in promoting the proliferation of HIG-82 chondrocytes than tortoiseshell. When tortoiseshell and antler were used together, the proliferation effects on both MC3T3-E1 osteoblasts and HIG-82 chondrocytes were superior to the effects of using tortoiseshell and antler separately. The result suggests that tortoiseshell and antler have a synergistic effect in alleviating osteoporosis and osteoarthritis. For instance, in the process of promoting osteoblast proliferation and calcium mineralization, antler, which is rich in collagen, calcium carbonate, and calcium phosphate, can significantly increase the bone density of osteoblasts, enhancing the effect of tortoiseshell in alleviating osteoporosis. Similarly, in the process of promoting chondrocyte proliferation, tortoiseshell, which contains animal collagen and various trace elements such as calcium, phosphorus, and zinc, can enhance the effect of antler in alleviating osteoarthritis. These results provide scientific evidence that GEJ can effectively treat osteoporosis and osteoarthritis.

Traditionally, Chinese medicine practitioners use GEJ to treat osteoporosis, degenerative arthritis, and other conditions. Clinical case reports also show its efficacy, although the mechanism is unclear. Ho et al. (2003) found that prolonged high-temperature decoction of GEJ causes the proteins to initially break down into many peptide fragments [14]. These fragments are further decomposed in the human digestive tract after oral administration, eventually becoming peptide fragments that can stimulate the proliferation of osteoblasts. These peptide fragments can enhance the absorption and utilization of calcium ions in the human body, and when used in combination with calcium ions, they have a synergistic effect on the proliferation and activation of osteoblasts [14]. Our study further found that tortoiseshell and antler contain various amino acids that can combine to form different bioactive peptides. Components from tortoiseshell can effectively promote the proliferation of osteoblasts and calcium mineralization, thereby effectively improving osteoporosis. Meanwhile, components from antler can effectively promote the proliferation of chondrocytes, thereby effectively improving degenerative knee arthritis.

Oxidative stress is closely related to osteoporosis and osteoarthritis [15]. Oxidative stress damage is closely associated with the accumulation of free radicals and reactive oxygen species (ROS). Due to environmental factors and unhealthy lifestyles, excessive free radicals and ROS may accumulate in the body, leading to redox imbalance and oxidative stress damage, which results in the occurrence of osteoporosis and osteoarthritis. As the body ages, the antioxidant defense becomes insufficient, increasing the amount of ROS and causing oxidative stress. Oxidative stress may trigger bone loss diseases such as osteoporosis. Osteoporosis is a typical bone disease and a chronic, hidden, age-related disease characterized by low bone density and fragile bones. Some cell studies and animal experiments have shown that free radicals affect osteoblast generation, osteoblast apoptosis, and bone resorption [16]. This is due to oxidative stress-related pathways in osteoporosis, such as ROS activation antagonizing the Wnt pathway, which is unfavorable for bone formation [17]. Osteoarthritis is the most common degenerative joint disease, leading to irreversible structural and functional changes in the joints, and is a major cause of disability and reduced life expectancy in the elderly population. Despite the high prevalence of osteoarthritis, there are no disease-modifying drugs available for its treatment. When oxidative stress results from an imbalance between ROS production and the antioxidant defense system, it induces inflammatory mediators such as interleukin-1β (IL-1β), tumor necrosis factor-α (TNF-α), and interleukin-6 (IL-6) to be highly upregulated in osteoarthritis joints, inducing ROS production and the expression of matrix-degrading proteases, leading to chondrocyte apoptosis and joint dysfunction [18].

Our research indicates that tortoiseshell and antler possess excellent antioxidant capacity, which we believe may be related to the amino acid composition of their active peptides. Our results in Figure 4B show that bioactive peptides from tortoiseshell, antler, and their combination exhibit different antioxidant capacities. In our future experiments, we hope to conduct an in-depth study on why the combination of tortoiseshell and antler and their active peptides has a better effect on both antioxidant capacity and cell viability than when used alone. This experiment only uses the MC3T3-E1 osteoblast and HIG-82 chondrocyte cell models to explore the therapeutic potential of bioactive peptides derived from natural products of tortoiseshell and antler in alleviating osteoporosis and osteoarthritis. In this study, we can only propose the possible mechanisms of action for the active peptides of tortoiseshell and antler in alleviating osteoporosis and degenerative knee osteoarthritis. To further confirm that the combination of tortoiseshell and antler and their active peptides has a better relieving effect on osteoporosis and degenerative knee osteoarthritis, we believe that using mouse models of osteoporosis or degenerative knee osteoarthritis or conducting clinical human trials should be our future research work.

## 4. Materials and Methods

### 4.1. Fingerprint Analysis and Preparation of Soluble Peptides

This study mainly collaborates with Sun Ten Pharmaceutical Company, which provides standard samples of tortoiseshell, antler, and the combination of tortoiseshell and antler, as well as bioactive peptides from GJ, tortoiseshell, and antler gelatin. They utilize liquid chromatography–mass spectrometry (LC/MS) to help establish the component fingerprint of tortoiseshell, antler, and the combination of tortoiseshell and antler and analyze the differences in amino acid and peptide fingerprints in the samples.

The preparation of samples (extraction solution): We precisely weigh 0.22 g of the test sample, mix 50 mL, 20 mL and 20 mL of 0.1% formic acid with ultrasonic oscillation at room temperature (about 25 °C) for 30 min (approximately equivalent to the concentration of the sample in the elution solution), and then let it cool. We take the supernatant and filter it with a 0.22 µm filter membrane (PVDF, Millipore millex-GV) to obtain the sample extraction solution.

Analysis conditions: amino acid fingerprint analysis: Shimadzu LCMS-2020 system liquid chromatography mass spectrometer.

- Controller: BM-20A.
- Pressurization system (pump): LC-20AD.
- Degassing system (degasser): DGU-20A.
- Sampling system (autosampler): SIL-20AC.
- Column oven: CTO-20A.
- Photodiode array detector: SPD-M20A.
- Mass detector: LCMS-2020.
- Pre-column: LichrospherRP-18 endcapped (5 μm, 4.0 × 10 mm, Merck).
- Analytical column: Intrada Amino Acid (3 μm, 3 × 50 mm, Imtakt).
- Column temperature: 35 °C.
- Injection volume: 10 μL.
- Moving phase: 100 mM HCOONH4; 0.1% HCOOHinCH3CN(1→1000).
- Flow rate: 0.6 mL/min.
- Analysis time: 13 min.
- Residence time: Ala: ~5.4 min, Glu: ~5.6 min, Leu: ~3.5 min, Ser: ~5.7 min, Arg: ~8.8 min, Gln: ~5.6 min, Lys: ~8.2 min, Thr: ~5.5 min, Asn: ~5.7 min, Gly: ~5.6 min, Met: ~4.0 min, Trp: ~3.2 min, Asp: ~5.7 min, His: ~7.8 min, Phe: ~3.2 min, Tyr: ~4.4 min, Cys: ~6.2 min, Ileu: ~3.8 min, Pro: ~4.4 min, Val: ~4.6 min.

Mass spectrometer detector: LCMS-2020.

- Ion source: electrospray ionization (ESI).
- Ion source interface voltage (interface voltage): select Tuning file.
- Nebulizing gas flow: 1.5 L/min.
- Drying gas flow: 20.00 L/min.
- Desolvation tube temperature (DL temp.): 300 °C.
- Heating module temperature (Heat block temp.): 500 °C.
- Scan mode: scan (+mode) m/z30-930.
- Selected ion monitoring mode: SIM (+mode).
- Amino acids and their m/z values: Ala: m/z 90.4, Glu: m/z 148, Leu: m/z 132, Ser: m/z 106.1, Arg: m/z 175.1, Gln: m/z 147, Lys: m/z 147, Thr: m/z 120.1, Asn: m/z 133, Gly: m/z 76.4, Met: m/z 150, Trp: m/z 205, Asp: m/z 134, His: m/z 156, Phe: m/z 166, Tyr: m/z 182, Cys: m/z 241, Ileu: m/z 132, Pro: m/z 116, Val: m/z 118.

### 4.2. DPPH Determination of Oxidative Free Radical Scavenging Ability

This experiment uses the DPPH free radical scavenging assay to respectively detect the antioxidant capacity of tortoiseshell, antler, and the combination of tortoiseshell and antler, as well as bioactive peptides from tortoiseshell and antler gelatin. The experimental procedure for the DPPH determination experiment, as reported previously [19], involved the following: A 100 μL DPPH (α,α-diphenyl-β-picrylhydrazyl, D9132) solution with a concentration of 1.5 mM/mL (Sigma-Aldrich Co., St. Louis, MO, USA) was mixed uniformly with tortoiseshell (0.01, 0.1, 1, 10.0 mg/L), antler (0.01, 0.1, 1, 10.0 mg/L), and the combination of tortoiseshell and antler (0.01, 0.1, 1, 10.0 mg/L), as well as bioactive peptides (0.02 mg/mL for each) from tortoiseshell and antler gelatin, respectively, and placed in a 96-well plate. The dosage ratio of tortoiseshell and antler in the combination was 50% each. The mixture was left to stand at room temperature for 30 min. The color change at a wavelength of 517 nm was recorded using a microplate reader (μQuant, Biotek Instruments, Inc., Winooski, VT, USA) and compared with the standard L-ascorbic acid (L-AA) (A5960, Sigma-Aldrich Co., St. Louis, MO, USA) to evaluate antioxidant capacity. According to the study by Gyamfi et al. (1999), the percentage of DPPH radical scavenging activity was calculated as follows: DPPH = (1 − (absorbance of sample/absorbance of standard L-ascorbic acid)) × 100. The lighter the color after adding DPPH to tortoiseshell, antler, and the combination of tortoiseshell and antler, as well as bioactive peptides from tortoiseshell and antler gelatin, the stronger the sample’s ability to capture DPPH free radicals. This result indicates that the added samples have the ability to capture DPPH free radicals; in other words, the better the antioxidant capacity of the added samples.

### 4.3. Cell Culture of MC3T3-E1 Osteoblasts and HIG-82 Chondrocytes

MC3T3-E1 osteoblasts and HIG-82 chondrocytes were purchased from the Bioresource Collection and Research Center (Hsinchu, Taiwan). The experimental procedure for the cell culture of MC3T3-E1 osteoblasts and HIG-82 chondrocytes, as reported previously [5], was as follows: The cells were incubated at 34 °C in a 5% CO_2_ atmosphere. To evaluate the cell proliferation potential of tortoiseshell, antler, and the combination of tortoiseshell and antler, as well as bioactive peptides from tortoiseshell and antler gelatin, MC3T3-E1 osteoblasts and HIG-82 chondrocytes were seeded in a 24-well plate at a density of 4 × 10^4^ cells per well. Subsequently, these cells were incubated with tortoiseshell (0.01, 0.1, 1, 10.0 mg/L), antler (0.01, 0.1, 1, 10.0 mg/L), and the combination of tortoiseshell and antler (0.01, 0.1, 1, 10.0 mg/L), as well as bioactive peptides (0.02 mg/mL for each) from tortoiseshell and antler gelatin, respectively, and placed in a 96-well plate. The dosage ratio of tortoiseshell and antler in the combination was 50% each, the concentrations of which are specified, for 24 h. Afterward, the cells were manually counted using MTT assay.

### 4.4. MTT Assay of Surviving MC3T3-E1 Osteoblasts and HIG-82 Chondrocytes

This study used (3-(4,5-dimethylthiazol-2-yl)-2,5-diphenyltetrazolium bromide) (MTT, M5655, Sigma-Aldrich) to respectively assess the MC3T3-E1 osteoblast and HIG-82 chondrocyte viability of tortoiseshell (0.01, 0.1, 1, 10.0 mg/L), antler (0.01, 0.1, 1, 10.0 mg/L), and the combination of tortoiseshell and antler (0.01, 0.1, 1, 10.0 mg/L), as well as bioactive peptides (0.02 mg/mL for each) from tortoiseshell and antler gelatin, respectively. The principle of the MTT assay mainly involves the action of succinate dehydrogenase in the mitochondria of cells, which converts the tetrazolium component in MTT into the blue product MTT formazan. The MTT formazan is then dissolved in DMSO for color development, and absorbance measurement is used to evaluate the cell viability of surviving MC3T3-E1 osteoblasts and HIG-82 chondrocytes. The experimental procedure for the MTT assay experiment, as reported previously [19], involved the following: After respectively treating MC3T3-E1 osteoblasts or HIG-82 chondrocytes with tortoiseshell, antler, and the combination of tortoiseshell and antler, as well as bioactive peptides from tortoiseshell and antler gelatin, 0.5 mg/mL MTT was added to the culture medium. Absorbance was read at optical density (OD) at 570 nm using an enzyme-linked immunosorbent assay (ELISA) reader (μQuant, Biotek Intruments, Inc., VT, USA).

### 4.5. Mineralization Assay of Surviving MC3T3-E1 Osteoblasts by Alizarin Red S Staining

The experimental procedure for the MTT assay experiment, as reported previously [20], involved the following: MC3T3-E1 osteoblasts, slowly rinsed by precooled PBS (2–3 times) to avoid the loss of cells, were fixed in an appropriate volume of 70% ethanol for 45 min, and then the ethanol was discarded. Next, MC3T3-E1 osteoblasts with treatments of tortoiseshell (50, 100 μg/mL), antler (50, 100 μg/mL), and the combination of tortoiseshell and antler (25 + 25, 50 + 50 μg/mL) in each well were stained for 10 min with a solution of 1 mL of 40 mM alizarin red S (Sigma) at pH 4.2 in deionized water at room temperature. With the staining solution being discarded, cells were then rinsed in deionized water three times and photographed under an inverted microscope. The amount of calcium mineral deposits was determined by dissolving cell-bound alizarin red staining in 10% acetic acid and the absorbance at 540 nm was measured by an ELISA reader (Bio-tek). Alizarin red S intensity relative to the control treatment was calculated after normalization to the total protein content.

### 4.6. Statistics and Analysis

We used a one-way analysis of variance (ANOVA) to evaluate the differences between groups. If a significant F-value was obtained, the Student–Newman–Keuls multiple-comparison post hoc test was performed. A *p*-value of less than 0.05 was considered significant, even if there was a notable error between groups.

## 5. Conclusions

Our research results indicated that the combined use of tortoiseshell and antler exhibited the best antioxidant capacity. Compared with antler treatment alone, tortoiseshell showed higher cell viability in MC3T3-E1 mouse osteoblasts but lower cell viability in HIG-82 chondrocytes. On the contrary, antler treatment alone has better antioxidant capacity and higher cell viability in HIG-82 chondrocytes than tortoiseshell treatment alone. Furthermore, soluble peptides from the combination of tortoiseshell and antler have better antioxidant capacity and cell viability in HIG-82 chondrocytes. Soluble peptides from antler gelatin alone also exhibit better antioxidant capacity and cell viability in HIG-82 chondrocytes compared to those from tortoiseshell; but soluble peptides from tortoiseshell treatment have a better cell viability of MC3T3-E1 osteoblasts than the soluble peptides from antler treatment at the same concentration. Our findings suggest that antler gelatin should have a better alleviating effect on degenerative joint diseases, while tortoiseshell gelatin should have a better alleviating effect on osteoporosis. When tortoiseshell and antler gelatin are used in combination, their antioxidant effects and efficacy in alleviating degenerative joint and osteoporosis should be superior compared to using either alone. To confirm our suggestion, animal and clinical trials should be necessary research work for the future.

## Data Availability

The data are confidential.

## References

[1] Liao J.A., Yeh Y.C., Chang Z.Y. (2022). The efficacy and safety of traditional Chinese medicine Guilu Erxian Jiao in the treatment of knee osteoarthritis: A systematic review and meta-analysis. Complement. Ther. Clin. Pract..

[2] Wu M.H., Lee T.H., Lee H.P., Li T.M., Lee I.T., Shieh P.C., Tang C.H. (2017). Kuei-Lu-Er-Xian-Jiao extract enhances BMP-2 production in osteoblasts. BioMedicine.

[3] Chou Y.J., Chuu J.J., Peng Y.J., Cheng Y.H., Chang C.H., Chang C.M., Liu H.W. (2018). The potent anti-inflammatory effect of Guilu Erxian Glue extracts remedy joint pain and ameliorate the progression of osteoarthritis in mice. J. Orthop. Surg. Res..

[4] Tsai C.C., Chou Y.Y., Chen Y.M., Tang Y.J., Ho H.C., Chen D.Y. (2014). Effect of the herbal drug guilu erxian jiao on muscle strength, articular pain, and disability in elderly men with knee osteoarthritis. Evid.-Based Complement. Altern. Med. Ecam.

[5] Lien C.Y., Lu C.W., Lin Y.H., Wu W.J., Hsu C.H., Chuang T.Y., Lin K.F., Chuang W.C., Lee M.C., Wu C.H. (2021). Chinese Herbal Medicine, Guilu Erxian Glue, as Alternative Medicine for Adverse Side Effects of Chemotherapy in Doxorubicin-Treated Cell and Mouse Models. Evid.-Based Complement. Altern. Med. Ecam.

[6] Li L.Q., Baibado J.T., Shen Q., Cheung H.Y. (2017). Determination of the authenticity of plastron-derived functional foods based on amino acid profiles analysed by MEKC. J. Chromatogr. B Anal. Technol. Biomed. Life Sci..

[7] Gu Y., Lu X., Jiang G., Deng J., Tang S., Li S. (2007). Comparison of the nourishing-yin functions of different types of tortoise shell. Lishizhen Med. Mater. Med. Res..

[8] Li X., Xie X., Huang C., Zhong Y., Li Y., Zhou J., Chen D. (2007). Repairing of oxidative damage to mesenchymal stem cell in rats and anti-lipid peroxidation by Plastrum testudinis ethanolic extract. Chin. Tradit. Herb. Drugs.

[9] Xie X., Li X., Zhong Y., Huang C., Du S., Li Y., Chen D. (2006). Study of antioxidant activities of Plastrum testudinis in Vitro. China Pharm..

[10] Li Y., Cui X., Chen D., Du S., Li H., Zhou J. (2003). Effect of tortoises shell on neural stem cell following injuried spinal cord. Chin. J. Neuroanat..

[11] Cheung H.Y., Cheung C.S. (1998). Nutritive value of plastron extracts and its effects on the differentiation of cancer cell. Hong Kong Pharm. J..

[12] Luo D., Li H., Li X., Zhou J., Du F., Wang L. (2008). Determination of collagen in tortoise shell using HPLC with phenyl isothiocyanate derivatization. Chin. Tradit. Herb. Drugs.

[13] Wu F., Li H., Jin L., Li X., Ma Y., You J., Li S., Xu Y. (2013). Deer antler base as a traditional Chinese medicine: A review of its traditional uses, chemistry and pharmacology. J. Ethnopharmacol..

[14] Ho T.J., Lin J.H., Lin S.Z., Tsai W.T., Wu J.R., Chen H.P. (2023). Isolation, Identification, and Characterization of Bioactive Peptides in Human Bone Cells from Tortoiseshell and Deer Antler Gelatin. Int. J. Mol. Sci..

[15] Riegger J., Schoppa A., Ruths L., Haffner-Luntzer M., Ignatius A. (2023). Oxidative stress as a key modulator of cell fate decision in osteoarthritis and osteoporosis: A narrative review. Cell. Mol. Biol. Lett..

[16] Almeida M., O’Brien C.A. (2013). Basic biology of skeletal aging: Role of stress response pathways. J. Gerontol. A Biol. Sci. Med. Sci..

[17] Almeida M., Han L., Martin-Millan M., O’Brien C.A., Manolagas S.C. (2007). Oxidative stress antagonizes Wnt signaling in osteoblast precursors by diverting beta-catenin from T cell factor- to forkhead box O-mediated transcription. J. Biol. Chem..

[18] Lepetsos P., Papavassiliou A.G. (2016). ROS/oxidative stress signaling in osteoarthritis. Biochim. Biophys. Acta.

[19] Wang C.H., Chung K.T., Su L.Y., Wu W.J., Wang P.H., Lee M.C., Shen S.C., Wu C.H. (2024). Chinese Herbal Medicines as Natural Alternative Products to Antibiotics in Weaned Piglets through Intestinal Microbiota Regulation. Int. J. Mol. Sci..

[20] Bernar A., Gebetsberger J.V., Bauer M., Streif W., Schirmer M. (2022). Optimization of the Alizarin Red S Assay by Enhancing Mineralization of Osteoblasts. Int. J. Mol. Sci..

